# Lumbar Epidural Cavernous Hemangioma: A Case Report and Review of the Literature

**DOI:** 10.7759/cureus.33677

**Published:** 2023-01-12

**Authors:** Jenny C Kienzler, Teresa E Hidalgo, Werner Wichmann, Alejandra Magagna-Poveda, Javier Fandino

**Affiliations:** 1 Neurosurgery, Kantonsspital Aarau, Aarau, CHE; 2 Pediatric Neurosurgery, New York University Langone Health, New York, USA; 3 Radiology and Neuroradiology, RNR Radiologie und Neuroradiolgie am Glattzentrum, Wallisellen, CHE; 4 Pathology, Kantonsspital Aarau, Aarau, CHE; 5 Neurosurgery, Hirslanden Medical Center Aarau and Zurich, Aarau, CHE

**Keywords:** epidural cavernous hemangioma, radiculopathy, lumbar, spinal, cavernoma

## Abstract

Pure epidural cavernous hemangioma (ECH) of the spine are rare and account for only 4% of all epidural spinal lesions. We report a case of epidural cavernoma at L3/4 presenting with L4 radiculopathy. Radiological, intraoperative findings and histopathology are presented.

We present the case of a 56-year-old man who was admitted with a right L4 radiculopathy including an M4 paresis of the right leg, hypoesthesia L4, and radicular pain. Magnetic resonance imaging (MRI) confirmed an extradural lesion L3/4 partially expanding into the right intervertebral foramen. The lesion had a heterogeneous signal, isointense on T1-weighted and hyperintense on proton density (PD) and T2-weighted images. At surgery, an epidural, ovoid, gray-red, soft mass, lightly adherent to the dura and extending to the right L4 foramen was observed. Findings in the histological examination indicated a cavernous hemangioma without signs of hemorrhage. Symptoms and paresis improved rapidly after surgery. The follow-up MRI showed complete resection of the lesion with no signs of radicular compression.

Spinal ECH should be considered as a cause of chronic lumbar radiculopathy with atypical radiological findings. Early diagnosis and total removal of the spinal ECH might prevent hemorrhage and neurological deficits. Fewer than 50 cases of lumbar epidural spinal hemangioma have been reported until today, and our case report is adding valuable knowledge to the existing literature.

## Introduction

Cavernous hemangioma is also known as cavernous angioma, cavernoma and cavernous malformation [1]. It is a rare vascular malformation that is sporadic or familial and can occur anywhere in the central nervous system or other organ systems in the body (liver, spleen, kidney, heart, and skin) [2]. Histologically, the lesion is characterized by grossly dilated vascular spaces, lined by a layer of endothelium, lack of mature vessel wall angioarchitecture, and features of chronic hemorrhage in adjacent parenchyma [3].

The incidence of cavernous hemangioma in the spine has been reported to be 0.22 cases/million/year, accounting for 5-12% of spinal vascular lesions [4]. About half of cavernous hemangiomas are extradural [5,6]. Most commonly, cavernous hemangioma occurs as a primary lesion in the vertebral body with variable secondary extension into the epidural space. Pure epidural cavernous hemangioma (ECH) of the spine account for only 4% of all epidural spinal tumors [4,7-15].

Presentation of spinal ECH is most common in the thoracic spine in approximately 60% of cases. Other locations include the cervical and lumbar spine in approximately 30% and 10-16%, respectively [5,16,17] Neurological deficits due to spinal ECH occur due to compression of a nerve root [18,19] or the spinal cord [7].

Cavernoma is embryologically supposed to be of endothelial cell origin, and because of that an extension of the epidural mass into the neuroforamen is possible [20]. Other common theories are that cavernous hemangiomas originate from dysplastic blood vessel progenitors or that they might develop from gradually increasing telangiectasias [21]. Furthermore, some malformations are not congenital and were reported as de novo cavernomatous lesions [22]. The exact mechanism remains unknown, but for example, trauma-induced is considered to be a potential cause of spinal cavernous hemangioma [23,24]. Associations of spinal ECH with cutaneous vascular lesions were described [25]. In this type of vascular lesion, genetic factors (CCM1 and CCM2 genes) have a well-known influence [26]. Other authors reported on spinal cavernomas associated with previous irradiation [27-30]. 

The clinical presentation of spinal ECH may be slow and intermittent in non-hemorrhagic cases [5,8] or present with acute pain and neurological deficits if bleeding occurs. Three different phenotypes of presentation have been described in the literature: slow seeping of blood from the cavernous malformation, and acute intralesional, or extralesional bleeding [10]. We present the clinical, radiological, and histological findings of a patient with an ECH in the lumbar spine, and conducted a literature review.

## Case presentation

History

A 56-year-old European man was admitted with symptoms of a right L4 radiculopathy including progressive gait disturbances without back pain. The symptoms were progressive during the last six months prior to admission. The neurological examination revealed a right M4 quadriceps paresis, as well as a right L4 hypoesthesia and dysesthesia and a positive straight leg raise test. No bowel or bladder disturbances were documented. The patient had other comorbidities: Coronary artery disease, and had undergone prior bypass surgery, familial hypercholesterolemia, and chronic inflammatory demyelinating polyneuropathy. Prior to surgery, he was prescribed Aspirin Cardio 100 mg, Atorvastatin 40 mg, and Ezetimibe 10 mg. 

Radiological findings

**Figure 1:**
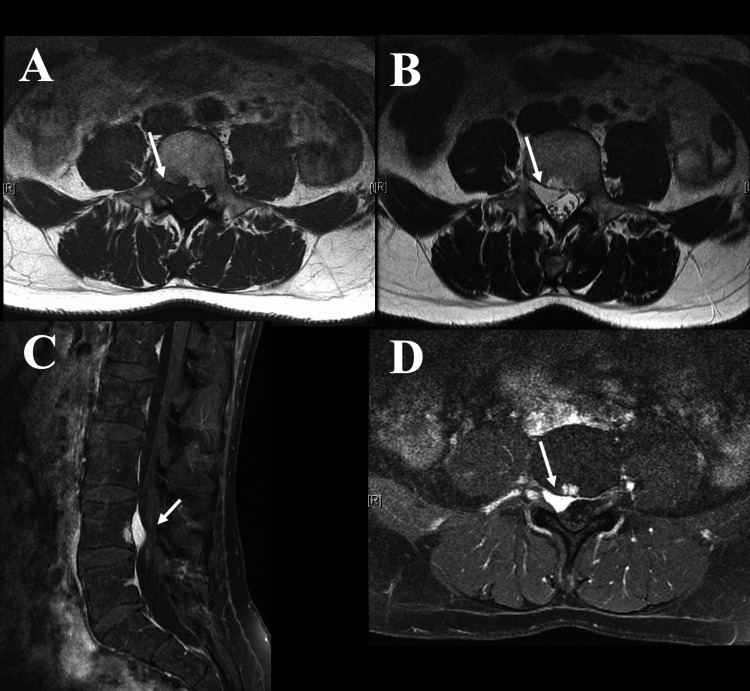
Preoperative MRI evaluation. Preoperative axial T1-weighted (A) and axial T2-weighted (B) MRI scans showing an extradural lesion compressing the nerve root L4. Contrast-enhanced T1 weighted sagittal (C) and axial (D) MRI scans demonstrates homogenous enhancement of the lesion. As differential diagnosis other pathologies such as lymphoma, leucaemic infiltration, plasmocytoma, and metastasis of an unknown primary tumor were considered.

The lumbar MRI showed an extradural lesion L3/L4 ventral to the cauda equina, compressing the right L4 nerve root at the recessus and partially expanding into the right L4 intervertebral foramen. The lesion surrounded the nerve root without any visible widening of the neuroforamen. Although there was some contrast enhancement at the vertebral body of L4, no clear affection of the adjacent vertebral bodies was visible on MRI and additional CT scan. The dumbbell-shaped, smooth lesion had an isointense signal on T1-weighted images and was hyperintense on PD and T2-weighted images (Figure 1A, 1B). A homogenous contrast enhancement on T1 images was observed (Figure 1C, 1D). These findings were preoperatively interpreted as a lymphoma rather than a disc herniation. 

Intraoperative findings

The patient underwent a microsurgical right interlaminectomy L3/4. Intraoperatively, an epidural ovoid and gray-red soft mass, lightly adherent to the dura, was found. The lesion extended into the right L4 foramen, compressing the right L4 nerve root. There were no signs of epidural hematoma. Under continuous bipolar coagulation and dissection, a microsurgical gross total en bloc resection of the lesion could be achieved. 

Histological findings

**Figure 2:**
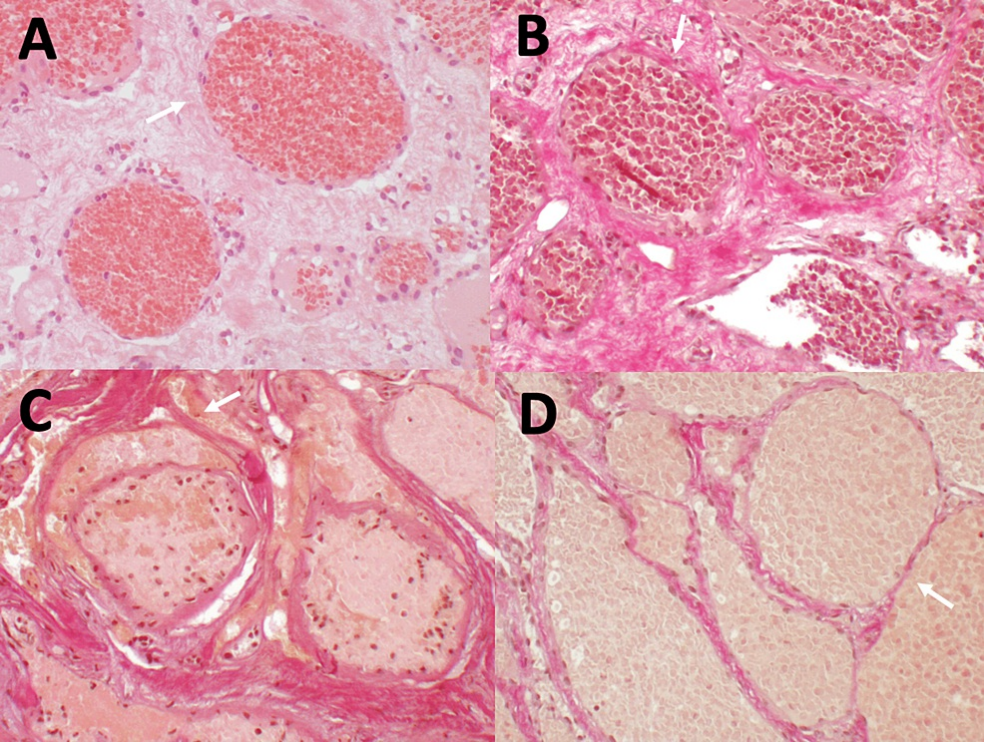
Results of histopathological examination. Microsections show a tissue composed of tightly packed collection of variably thickened and hyalinized, dilated blood-filled vessels. The blood vessel walls are thin and consist of endothelium and a collagenous adventitia lacking elastic tissue (A: HE, 100x; B: EvG, 100x; C: EvG, 100x; D: EvG, 100x).

Findings in the histological examination indicated a hemangioma of the cavernous subtype without evident signs of hemorrhage. Hematoxylin and eosin-stained microsections showed tissue composed of a tightly packed collection of variably thickened and hyalinized, dilated, blood-filled vessels with no intervening spinal cord parenchyma. The blood vessel walls consisted of endothelium and a collagenous adventitia lacking elastic tissue and with little smooth muscle cell component, as shown on EvG-stained sections (Figure 2).

Postoperative course and follow-up

**Figure 3:**
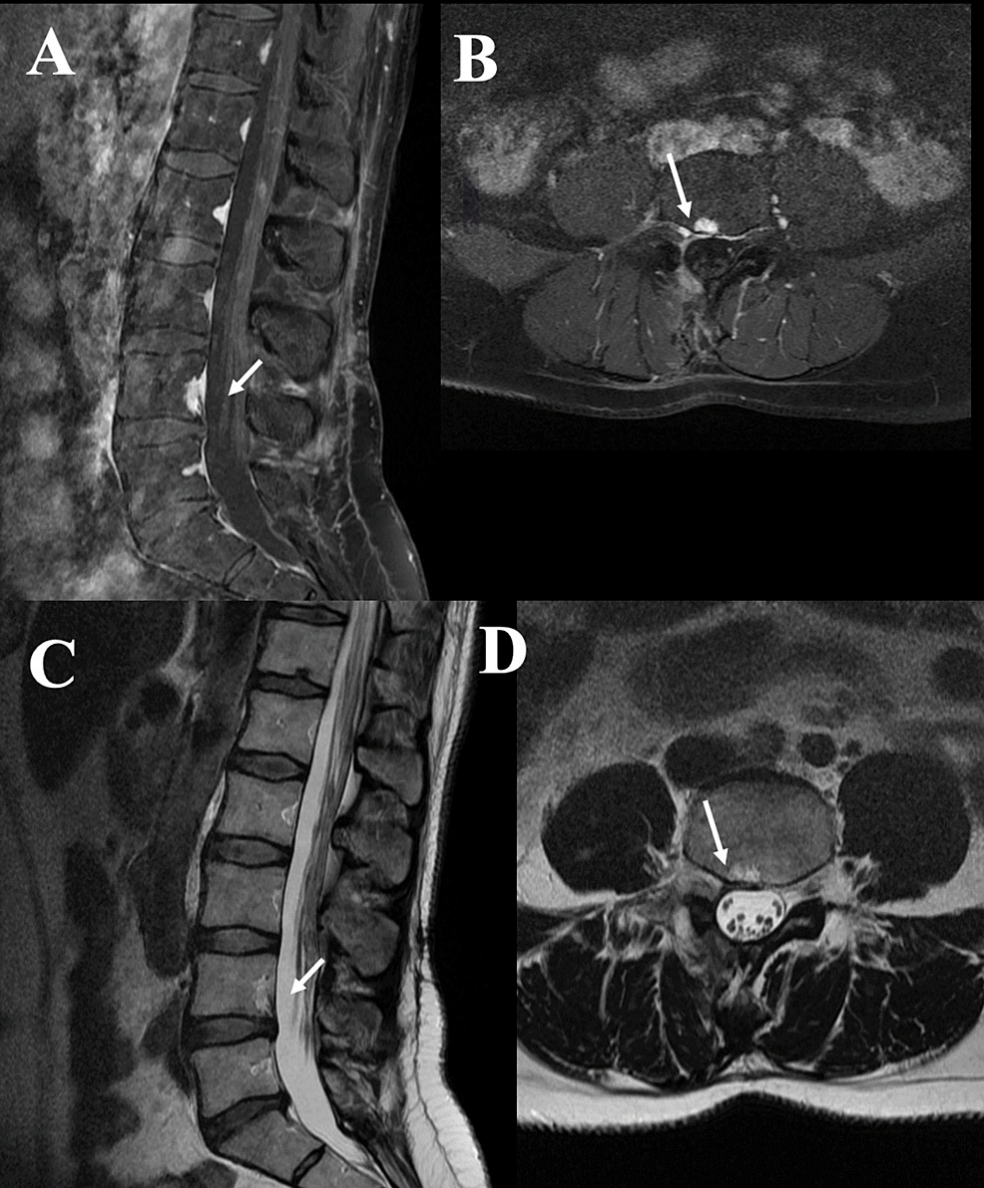
Postoperative radiological findings. Six months postoperative T1-weighted contrast enhanced sagittal (A) and axial (B) as well as T2-weighted sagittal (C) and axial (D) MRI scans demonstrated completely resection of the lesion and radicular decompression.

No intra- or postoperative complications occurred. Symptoms including paresis rapidly diminished after surgery. The follow-up MRI and CT six months after surgery showed complete resection of the lesion with no signs of radicular compression (Figure 3). 

Due to progressive symptoms of the patient's chronic inflammatory demyelinating polyneuropathy, without any recurrence of the radiculopathy, another MRI was performed 14 years after the initial surgery. The MRI revealed residual postoperative changes without unambiguous recurrence of the ECH, or progression of the intraosseus component. 

## Discussion

We present a case of a spinal lumbar ECH with radicular pain due to nerve root compression. Since the first description in 1929 by Globus and Doshay [23,31], approximately 200 cases of spinal hemangioma were reported [4,9,10,17,21,25,31-55]. Less than 50 cases of lumbar epidural spinal hemangioma have been reported until today, and therefore our case report is one of a few cases published in the literature over the past 50 years.

Clinical findings

Roughly 50% -70% of the cases occurred in women [12,44,56] and the age of onset varied between 30 and 60 years, with a peak around 40 years [57]. The size of the lesion has been described from a few millimeters to several centimeters [11]. Presentation at admission varies from no neurological deficit (10%) to radiculopathy (10%) and myelopathy (80%) [12,58]. Symptoms can be aggravated by trauma, heavy exercise, systemic infection, or pregnancy [45,59]. Aoyagi et al. evaluated 54 cases with spinal epidural cavernous hemangioma without primary origin in the vertebral bone [9]. The authors found, that most lesions were in the thoracic spine (80%) and on the dorsal side of the spinal cord (93%). The preference for this localization could be caused by a larger available epidural space and lower resistance in the posterior spinal canal [9,17,33]. The clinical course in this series was slowly progressive with myelopathy in 33% of the cases at onset and 83% at admission [9]. Cavernous hemangioma is a benign hamartomatous vascular anomaly, which increases slowly in volume and therefore can explain progressive clinical deterioration. The clinical course can be progressive or acute, similar to a growing epidural tumor. The progress depends on the biological behavior including the growth rate and location of the lesion [25].

In cases with a lumbar lesion, as in our case, patients present with lower back pain and radiculopathy caused by the extension of the cavernoma into the neuroforamen or the ventrolateral compartments, mimicking symptoms of a disc herniation [12,17,20,60-62]. A more rapid increase in the volume of the lesion can be caused by thrombotic venous occlusion [61], draining vein compression by an enlarged pregnant uterus [63-65], or due to neovascularization through an increase in estrogen levels, leading to a sudden clinical deterioration [66]. Other causes of acute symptoms are microhemorrhages within the lesion, extradural hemorrhage surrounding the lesion, hematomyelia, and subarachnoid hemorrhage [8,61,67,68]. Hemorrhage can be triggered by straining, intake of anticoagulants, or occur spontaneously [39]. In principle cavernomas located at the lumbar spine have a longer history of symptoms [69]. This may be caused by the fact that nerve roots can tolerate long-term soft tissue compression much better than the spinal cord. However, most patients with epidural cavernous hemangioma have a chronic clinical course with gradual worsening of symptoms over several months [39].

Neuroradiological appearance

According to the MRI findings, spinal epidural vascular lesions are categorized into four types [38]: 1) arteriovenous type with organized hematoma 2) venous type, 3) cavernous type and 4) cavernous type with hematoma. There are several MRI characteristics such as high-level intensity [but slightly less signal than cerebrospinal fluid (CSF)] on T2-weighted images, and low-level intensity or isointensity with homogenous contrast enhancement on T1-images [44]. Another characteristic MRI presentation is the so-called “wafting silk” [70], due to the sinusoidal vascular structure, as shown in our case [7,71-73]. The explanation for the signal on T2 weighted images could be the high content of stagnant blood and slow blood flow [70]. Intralesional hemorrhage can lead to hyperintensity in T1 and T2 images [17]. However, cavernous hemangiomas are angiographically occult except for vertebral body lesions and do not develop any large vascular supply or venous drainage [74,75]. Intramedullary cavernous malformations typically produce a high-level intensity ring in T2 weighted images (hemosiderin ring) which can be seen in epidural cavernomas as well [10,38,39]. Earlier studies have claimed, that the lack of a low-signal hemosiderin ring on both T1- and T2 weighted images is an important difference to intramedullary lesions [7,19,23,70,76-78]. This could be a result of the rapid absorption of blood products in the epidural space [7]. In our case, the lesion might not be purely but primarily epidural, because of contrast enhancement in the vertebral body and therefore possible origin of the cavernoma from the vertebral body with extension into the epidural space and the neuroforamen. Interestingly, all reported cases of ECH in the lumbar and sacral spine, show an anterior epidural space localization, close to the vertebral bone [10,17,19,38,39,70,79].

Differential diagnosis

For the differential diagnosis, radiological findings and clinical history are key points. The differential diagnosis of an EHC per se includes disc fragments, epidural fat, atypical schwannoma, an enlarged epidural venous plexus, lymphoma, sarcoidosis, chordoma, eosinophilic granuloma, multiple myeloma, meningioma and neurinoma [9,70,78,80]. In our case, the most likely differential diagnosis taking history and MRI findings into consideration were lymphoma, leukemic infiltration, plasmacytoma, and metastasis of an unknown primary tumor. 

Histological considerations

Cavernous hemangiomas may be mistaken macroscopically in the central nervous system for fresh, demarcated brain/spinal cord hemorrhages. Histologically, they appear as a tightly packed collection of variably thickened and hyalinized, dilated, vascular channels with no intervening spinal cord/brain parenchyma [23]. The blood vessel walls consist of endothelium and a collagenous adventitia lacking elastic tissue and little or no smooth muscle cell component [23,34,55,81,82], but may include foci of calcifications (or even ossifications) and can be thrombosed. Hemosiderin, which is almost universally present within intramedullary and intraparenchymal lesions, is much less abundant in vertebral and epidural lesions. However, epidural lesions possess far greater vascularity, which may provide for the more rapid removal of hemosiderin [83].

Surgical considerations

In case of clinical or radiological evidence of acute hemorrhage, immediate spinal decompression and hematoma evacuation should be performed to avoid further neurological deterioration [13,84,85]. Workup of spontaneous spinal epidural hemorrhage should mandatorily include MRI for evidence of spinal ECH, due to the significant risk of rebleeding and possible neurological worsening [22,74]. The risk of bleeding and rebleeding is comparable to supratentorial cavernomas and ranges from 0.25% to 0.7% per person-year [86]. Epidural cavernous hemangiomas are rather easy to resect [87] and should be removed en bloc [57]. Therefore, the indication for surgical treatment is given even in incidental cases to avoid neurological decline over time [88]. Nevertheless, severe intraoperative bleeding has been, reported during surgery for spinal ECH [61]. When the cavernoma encases the spinal nerve root, and dissection from the tumor mass is needed, intense bleeding can occur due to violation of the cavernoma [39]. The risk of hemorrhage and recurrence is increased by partial or subtotal resection [9]. Initial shrinkage of the tumor through extended low-bipolar coagulation can prevent massive bleeding and permits en bloc resection [89]. Radiotherapy and stereotactic radiosurgery (hypofractionated dose of 32 Gy in 4 fractions) [73] remain controversial and should be reserved for unresectable lesions or postoperative residuals [6,81,90,91]. A good recovery with an improved neurological condition and complete remission of symptoms can be achieved in most patients after total removal of the cavernoma and was observed in our patient [8,9,44,92]. Some authors suggested, that the most important prognostic factor, is the preoperative neurological status [39].

Due to a high incidence of multiple lesions, the whole neuraxis should be screened, since approximately 40% of patients with spinal cavernomas have cranial lesions [87,93]. The MRI screening of our patient did not unveil any intracranial lesions. 

## Conclusions

Spinal epidural cavernous hemangioma should be included in the differential diagnosis in patients with atypical radiological findings as a benign cause of chronic lumbar radiculopathy. Early diagnosis and total removal of the lesion should be achieved to avoid the risk of hemorrhage and consecutive neurological deficits. Surgical resection is the standard of care and resulted in an excellent outcome with complete and sustainable pain relief and no new neurological deficits. Due to the high incidence of combined presentation of spinal and cranial cavernomas, screening of neuraxis is recommended in patients presenting with spinal cavernomas. 
